# Assessing the Trend of the Trophic State of Lake Ladoga Based on Multi-Year (1997–2019) CMEMS GlobColour-Merged CHL-OC5 Satellite Observations

**DOI:** 10.3390/s20236881

**Published:** 2020-12-01

**Authors:** Augustine-Moses Gaavwase Gbagir, Alfred Colpaert

**Affiliations:** 1 School of Forest Sciences, University of Eastern Finland, Yliopistokatu 7, Borealis Building A 3rd Floor, 80101 Joensuu, Finland; 2 Department of Geographical and Historical Studies, University of Eastern Finland, Yliopistokatu 7, Metria-Building, P.O. Box 111, FI-80101 Joensuu, Finland; alfred.colpaert@uef.fi

**Keywords:** Lake Ladoga, CMEMS GlobColour CHL-OC5, eutrophication, water quality assessment, pulp and paper mill, climate change, ecological status, remote sensing, phytoplankton and chlorophyll-a, chemical wastewater pollution

## Abstract

The trophic state of Lake Ladoga was studied during the period 1997–2019, using the Copernicus Marine Environmental Monitoring Service (CMEMS) GlobColour-merged chlorophyll-a OC5 algorithm (GlobColour CHL-OC5) satellite observations. Lake Ladoga, in general, is mesotrophic but certain parts of the lake have been eutrophic since the 1960s due to the discharge of wastewater from industrial, urban, and agricultural sources. Since then, many ecological assessments of the Lake’s state have been made. These studies have indicated that various changes are taking place in the lake and continuous monitoring of the lake is essential to update the current knowledge of its state. The aim of this study was to assess the long-term trend in chl-a in Lake Ladoga. The results showed a gradual reduction in chl-a concentration, indicating a moderate improvement. Chl-a concentrations (minimum-maximum values) varied spatially. The shallow southern shores did not show any improvement while the situation in the north is much better. The shore areas around the functioning paper mill at Pitkäranta and city of Sortavala still show high chl-a values. These findings provide a general reference on the current trophic state of Lake Ladoga that could contribute to improve policy and management strategies. It is assumed that the present warming trend of surface water may result in phytoplankton growth increase, thus partly offsetting a decrease in nutrient load. Precipitation is thought to be increasing, but the influence on water quality is less clear. Future studies could assess the current chemical composition to determine the state of water quality of Lake Ladoga.

## 1. Introduction

**Figure 1:**
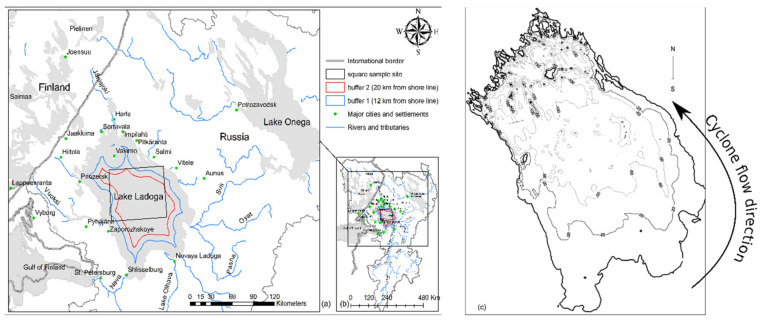
Shows Lake Ladoga, the study area. In (**a**), zoomed in with Ladoga in the center, Lake Onega (upper right), and Gulf of Finland (lower left corner), buffer 1, buffer 2, square are sample sites. In (**b**), Lake Ladoga drainage basin. In (**c**), is the bathymetric map of Lake Ladoga (image adapted and modified from Subetto et al. 1998 [20]).

Assessing the trophic state of Lake Ladoga is essential because of its past history of severe water pollution [1,2]. The history of the Great Lake Ladoga begins after the melting of the ice of the last glaciation, when Ladoga became separated from the so-called Yoldia Sea about 10,000 years ago [3]. The earliest drainage of the lake was to the Gulf of Finland in the direction of the region now known as the city of Vyborg through the threshold of Vetokallio near Heinjoki [3]. Post-glacial uplift, which was, and still is, faster in the north, tilted Lake Ladoga causing a transgression of its southern shallow shoreline, leading to the creation of the current Neva River connection about 3300 years ago [3]. For the same reason, about 9500 years ago, a connection was established from Lake Onega to Ladoga via the River Svir [3]. The birth of the Neva caused the surface of Ladoga to drop by about 12 m, producing the present shape of the lake [3]. The Lake was oligotrophic (mostly free from nutrients) before the 1950s and mesotrophic (moderate nutrients) in the 1970s but some areas of the lake (mostly coastal locations) have become eutrophic due to anthropogenic activities [1,4]. These anthropogenic activities were mainly industrial enterprises (e.g., pulp and paper mills, aluminum) and agriculture [1,5]. Consequently, the discharge of wastewater (containing high amounts of phosphorus and nitrogen) into the lake from these industries resulted in the degradation of water quality [1,2]. The lake has a large catchment (Figure 1), and thus receives huge amounts of water containing anthropogenic nutrients [2,6]. An important part of these waste waters discharge into the shallow Volkhov Bay (southern coastal area) which receives its waters from the Volkhov sub-catchment (West Ladoga), and Syas River (from the South) [5]. Also, extensive agricultural activities resulted in the washing of phosphorus and nitrogen into the lake [1,6]. It is well documented that the lake contains toxic chemical substances, pesticides, other harmful material as well as large amounts of sediment [5,6]. Eventually, Lake Ladoga flows through River Neva near St-Petersburg into the Gulf of Finland [7]. The lake is the main source of clean water not only for local communities, but also for the city of St. Petersburg (over five million inhabitants), thus there is much pressure to maintain good water quality. Also, the importance of the lake for recreational use is steadily increasing [6,8].

Thus, continuous monitoring of the ecological status and water quality of this large lake is essential to understand and assess changes in its environment. Monitoring activities have been on-going for several decades [1,3,5,6]. In this paper, ecological status means the assessment of water eutrophication, through changes in phytoplankton levels. The use of phytoplankton biomass and chlorophyll-a (chl-a) concentration is a common method employed to study ecological status in water bodies [9,10]. This method is successful because chl-a correlates well with phytoplankton biomass abundance [9,11] and is used as an indicator for eutrophication [10]. Currently, low-cost instruments exist for measuring in situ chl-a in water bodies [12,13,14]. On the other hand, the use of these low-cost instruments still rely on the use of expensive research vessels and time consuming field surveys. However, the development of remote sensing and satellite-based imagery serves a wider audience [10,15]. Although estimates of chl-a based on remote sensing are generally less accurate than laboratory measurements, the approach provides a cost-effective and sufficiently reliable regional picture of a phenomenon that can be used for environmental monitoring and management needs. By using remote sensing data, the amount of chl-a can be calculated using empirical formulas based on the correlation between the violet-green light reflected from the water surface and the measured amounts of chl-a [16]. In addition, remote sensing methods provide historical time series of observations, for example, Landsat satellite data have been available since 1972 and MODIS data since 2000. Pozdnyakov et al. [1] have used SeaWiFS satellite images to study the biochemical properties of Ladoga from 1998–2004. Pozdnyakov et al. [1], were also able to validate the consistency of remote sensing derived chl-a with in situ chl-a measurements in Lake Ladoga. Studies using temporal remote sensing data to study the ecological status of Lake Ladoga are quite few and none have been done after 2010 (e.g., Pozdnyakov et al. [1]). The complex nature of coastal and lake water bodies [excessive colored dissolved organic matter (CDOM) and mineral particles] makes it difficult to use remote sensing to assess chl-a concentrations (so called Case II water bodies), thus many empirical, local, algorithms have been developed [1]. Developing different algorithms to suit each local water type around the globe is a dilemma. Thus, the main goal of this study was to use a global remote sensing data product that has considered the complex nature of different water types. In this study, we utilized the merged chlorophyll-a (chl-a) product derived from SeaWiFS, MERIS, MODIS Aqua, VIIR, and OLCI satellite sensors (1997–2019) to characterize the general trend of chl-a in Lake Ladoga. The novelty in our approach is to use geostatistical analytics of remote sensing data to produce a historical and synoptic picture of the state of Lake Ladoga. We anticipate that the results of this long-term assessment will contribute to the current information on the ecological status of Lake Ladoga. This additional information may be beneficial to on-going policy frameworks and management strategies of Lake Ladoga.

## 2. Materials and Methods

### 2.1. Study Area

Lake Ladoga is the largest lake in Europe having a surface area of 17,765 km^2^ [17], with an average depth of 48.3 m [17] and maximum depth of 230 m (Figure 1) [6,17]. The lake has a volume of 858 km^3^ [17], a water retention time of 12 years, and a coastline of 1570 km [17,18]. The lake has a total catchment area of 282,200 km^2^, 20% of the area is located in Finland [6] (Figure 1b). The deepest parts of the lake are in the North (60–200 m), and the shallowest parts (10–50 m) in the southern parts of the basin [4]. The northern shores of the lake are rugged and rocky, while the western, southern, and eastern shores are shallow with fine-grained sediments [4]. The East and South contain extensive sandy beaches as well [4]. Water from Finland flows via the River Vuoksi from the west into Lake Ladoga while from the East, the water from Lake Onega drain via the River Svir [4]. From the South, the waters from Lake Ilmen flow into Ladoga via the River Volkhov [4]. Also, the River Syas and dozens of smaller streams and rivers flow into Ladoga as well [5]. Eventually, Lake Ladoga flows through River Neva near St-Petersburg into the Gulf of Finland [7].

Mean air temperature in the area is +3.2 °C [18,19]. The coldest month (February) has a mean temperature of −8.8 °C, while the warmest month (July) has +16.3 °C [19]. The mean annual precipitation in the area is about 475 mm [19].

Along Lake Ladoga’s shores there are several cities which include: Sortavala, Priozersk, Shlisselburg, Novaya Ladoga, and Pitkäranta (Figure 1a), with a combined population of about 100,000 people. Also, there is a long history of industrial activity, mainly wood processing industries. Some closed pulp and paper mills were located near Priozersk, and Harlu, Läskelä. Currently, there are still functioning pulp mills in Pitkäranta in the North-East and near the town of Novaya Ladoga in Syasstroy (south). Lake Ladoga is inhabited by several endangered species, as the Ladoga Lake Salmon and the Ladoga Ringed Seal [18].

### 2.2. Data

We used the merged (4 km × 4 km) global monthly chlorophyll (chl-a) product (1997–2019) from GlobColour [21]. The merged chlorophyll-a OC5 algorithm product (CHL-OC5) is available from September 1997 to present and is updated annually [21]. The global monthly chl-a is a “Cloud Free” level four (L4) product created by merging data from four satellite sensors, the Sea-Viewing Wide Field-of-View Sensor (SeaWiFS), Medium Resolution Imaging Spectrometer (MERIS), Moderate Resolution Imaging Spectrometer (MODIS), Visible Infrared Imaging Radiometer Suite (VIIRS), and Ocean and Land Color Instrument (OLCI) [21]. The GlobColour products were downloaded from the Copernicus Marine Environmental Monitoring Service (CMEMS) website: http://marine.copernicus.eu/services-portfolio/access-to-products/.

CMEMS CHL-OC5 dataset has been created using the OC5-algorithm which has been shown to work very well for Case II water bodies and has been validated using a large number of global in situ data from sea and lake areas [21,22]. We tested the dataset for lake Ladoga using data from Letanskaya and Protopopova [23], giving a reasonble fit, the CHL-OC5 dataset seemed to overestimate chl-a values in the lower range (below 5 mg m^−3^), but for the higher ranges (over 15 mg m^−3^) the data was consistent with the in situ values. As our main interst was in the trend of water chlorophyl-a content and not in absolute values, we used the CMEMS CHL-OC5 dataset in our study, with the notion that the values are not exact values, but relative indicators derived from a global dataset.

The datasets are for the months of June, July, August, September, and October. The downloaded chl-a data was processed further using the SeaWiFS Data Analysis System (SeaDAS) software [24]. SeaDAS is a free and opensource software developed and maintained by Ocean Biology Processing Group (OBPG) [24]. In SeaDAS, the land and water mask were created, and the no data layer added. The appropriate vector data was added, and the final images exported and compiled using Inkscape [25].

Additional processing of the chl-a was done with R software (R Core Team, 2020, [26], with raster package [27], to extract the mean, minimum and maximum values of chl-a. The results were then written to Excel format with the R package writexl [28]. Also, for each year (1997–2019), we calculated the (seasonal) mean of means for the five months (June, July, August, September, and October). Hence, in this study we used season to represent the calculated mean of the five months. We calculated the mean over the whole lake as a unit and within three sub-sample areas (buffer 1, buffer 1, and central part of the lake). Buffers 1 and 2 were twelve and twenty kilometers, respectively, from the shoreline (Figure 1). When calculating the season means, we excluded 1997 (only September) in the trend analysis to prevent bias in the result.

## 3. Results

### 3.1. General Trend of Chl-a Concentration in Lake Ladoga: 1997–2019

**Figure 2:**
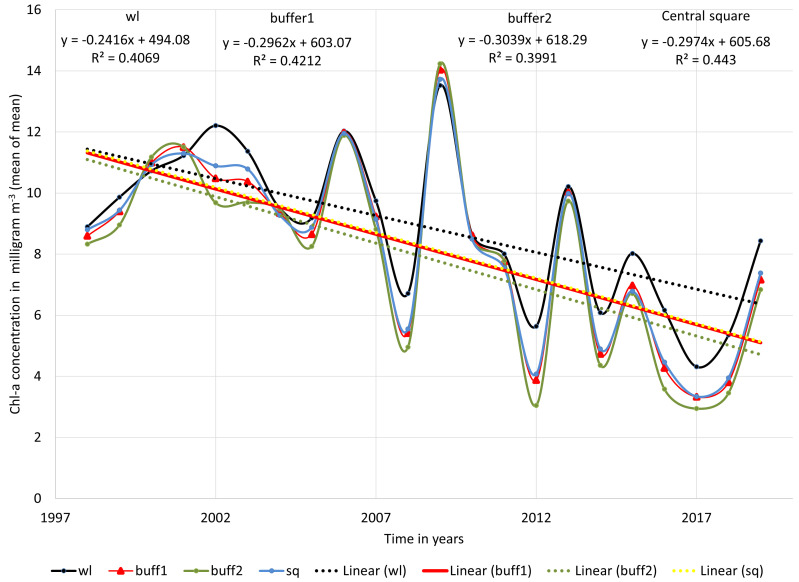
The average seasonal trend of chlorophyll-a concentration (mg m^−3^) 1998–2019. wl = whole Ladoga lake, buffer1 = 12 km from shore line, buffer2 = 20 km from shore line, and central square = the sample site in the middle of the lake.

The general trend of seasonal chl-a concentration in Lake Ladoga is negative (Figure 2, wl). The negative trend is gradual (slope = −0.12416 and R^2^ = 0.4069). This indicates a moderate improvement (decline in chl-a). In general, 2009 had the highest chl-a concentrations (>10 mg m^−3^) while the lowest value was measured in 2017 (<5 mg m^−3^). Similarly, the trend in the sub-sampled locations (s1, s2 and s3), was negative but non-significant ((buffer1: slope = −0.2962 and R^2^ = 0.4212); (buffer2: slope = −0.3039 and R^2^ = 0.3991); (center square: slope = −0.2974 and R^2^ = 0. 433)). Water surface temperature (SST) had no observable trend in the lake during the study period.

### 3.2. Spatial Distribution of Chl-a Concentration Across Lake Ladoga

**Figure 3:**
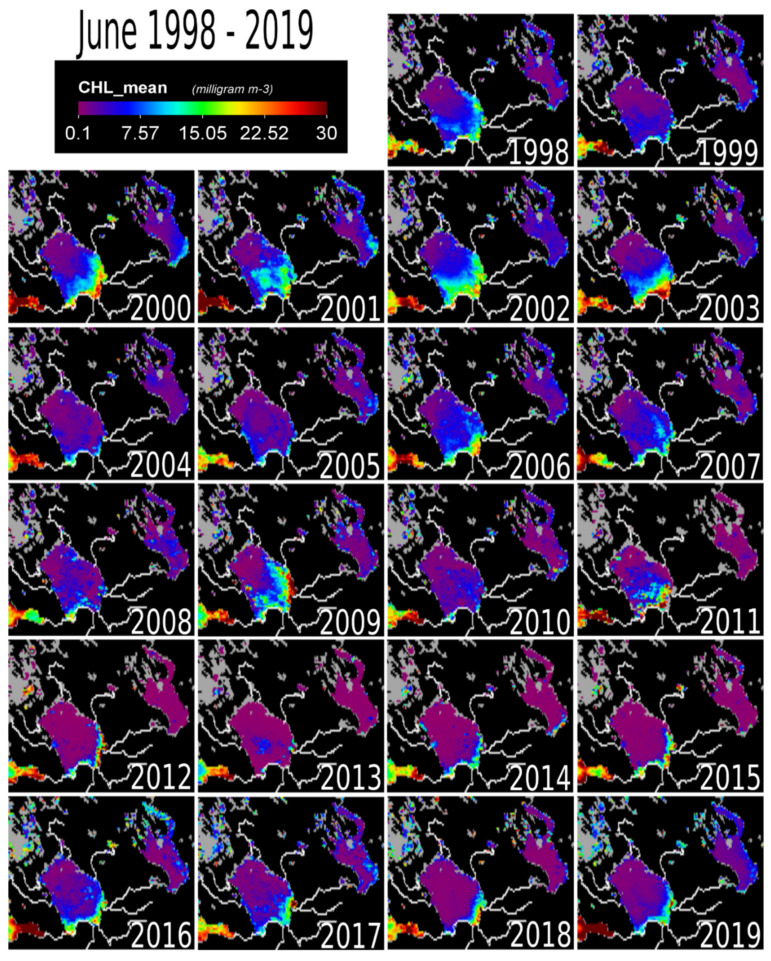
The map of chlorophyll-a concentration at Lake Ladoga for the month of June (1998–2019). Partially displayed as reference points are the Gulf of Finland (Southwest) and Lake Onega (Northeast).

**Figure 4:**
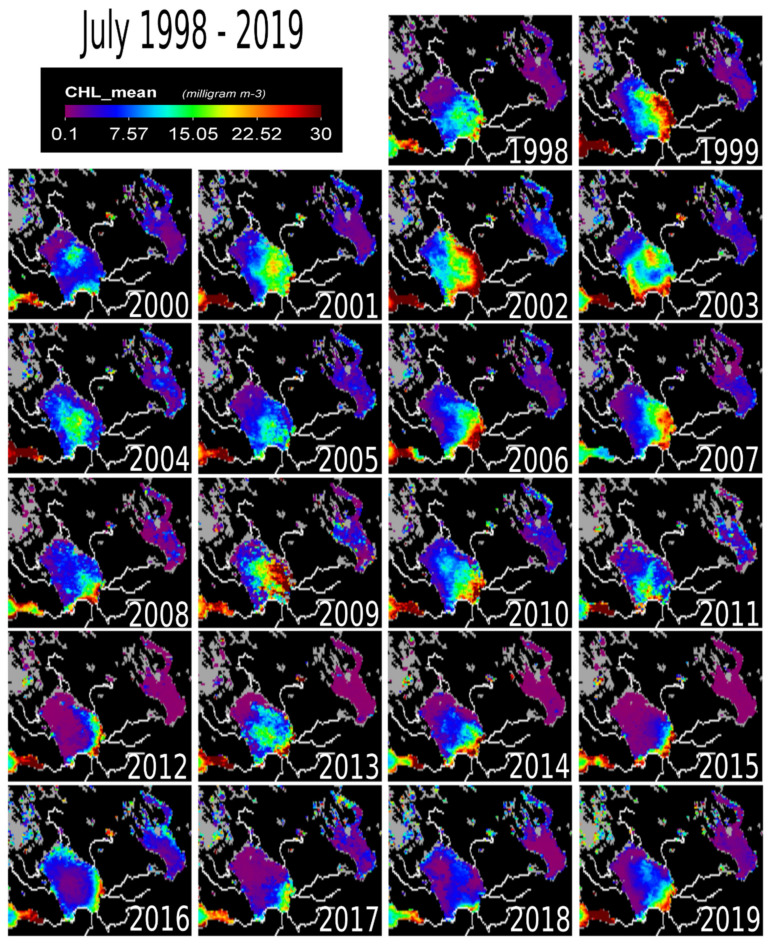
The map of chlorophyll-a concentration at Lake Ladoga for the month of July (1998–2019). Partially displayed as reference points are the Gulf of Finland (Southwest) and Lake Onega (Northeast).

**Figure 5:**
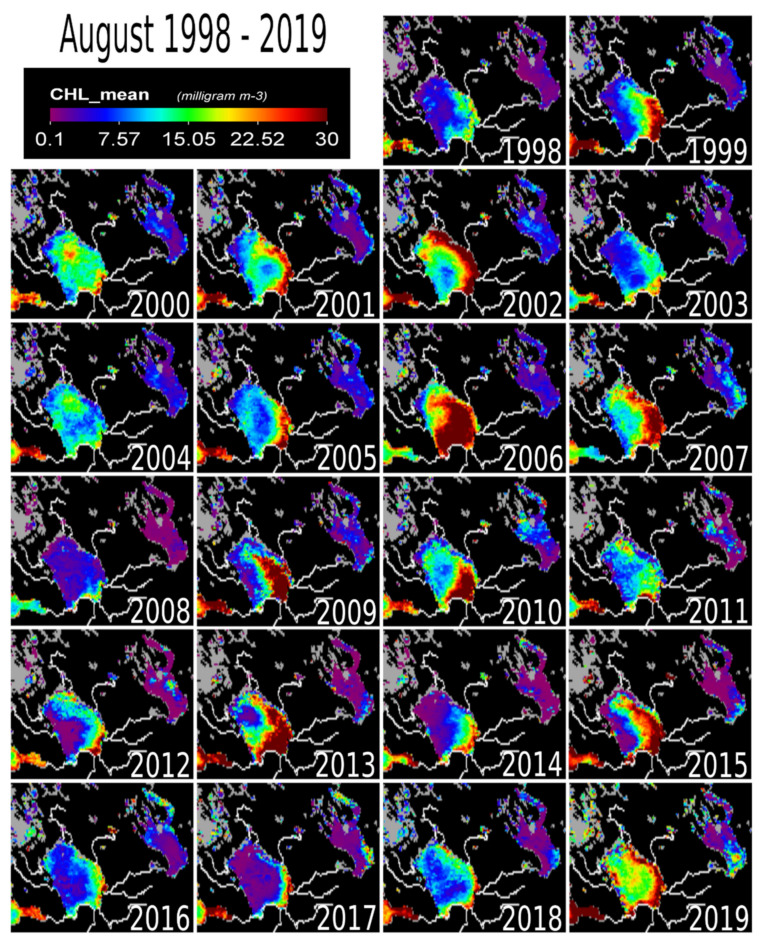
The map of chlorophyll-a concentration at Lake Ladoga for the month of August (1998–2019). Partially displayed as reference points are the Gulf of Finland (Southwest) and Lake Onega (Northeast).

**Figure 6:**
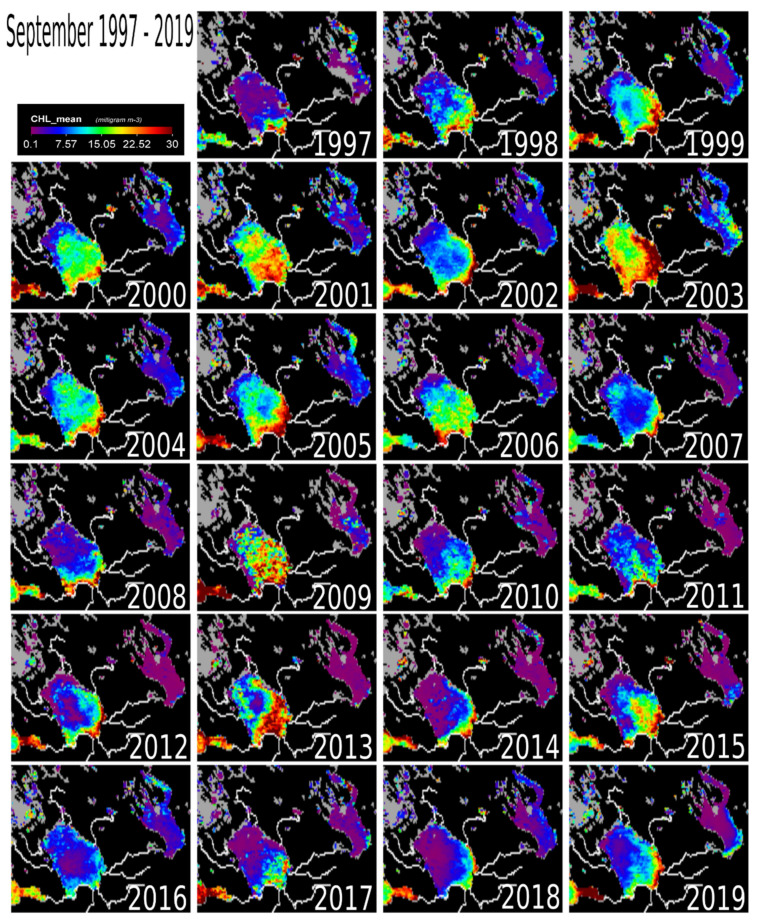
The map of chlorophyll-a concentration at Lake Ladoga for the month of September (1997–2019). Partially displayed as reference points are the Gulf of Finland (Southwest) and Lake Onega (Northeast).

**Figure 7:**
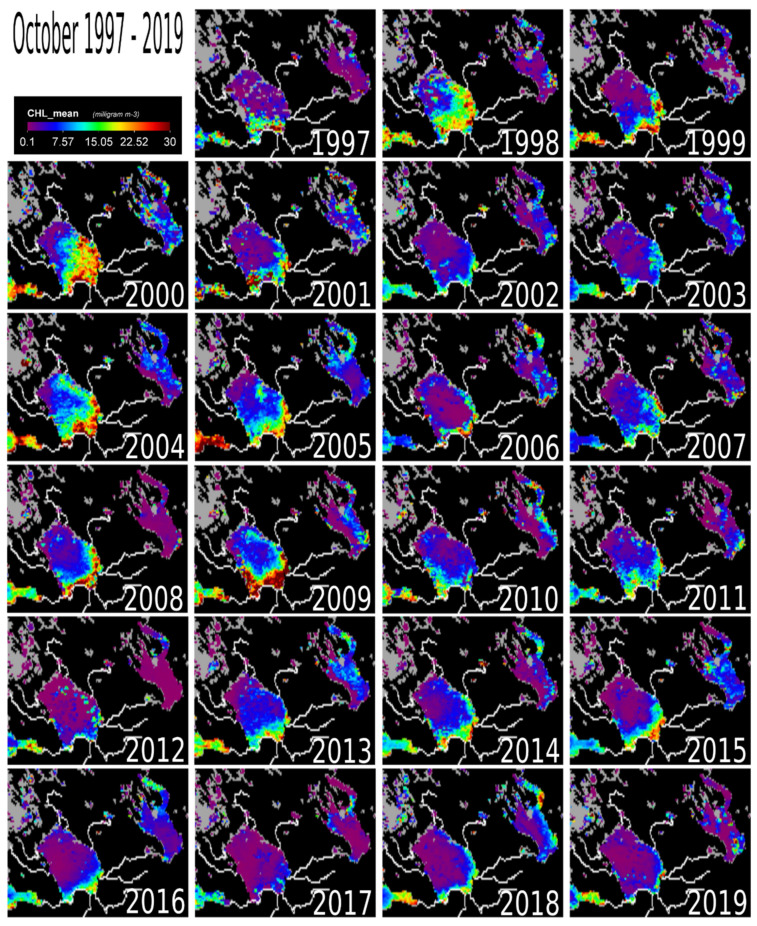
The map of chlorophyll-a concentration at Lake Ladoga for the month of October (1997–2019). Partially displayed as reference points are the Gulf of Finland (Southwest) and Lake Onega (Northeast).

Figure 3, Figure 4, Figure 5, Figure 6 and Figure 7 show the pixel-wise variability of chl-a concentration across the lake. For June, wide distribution of chl-a concentration (moderate to high values) was observed for 1998, 2000, 2002, 2003, 2006 and 2009 (Figure 3). The high values were along the shallow southern shoreline and moderate values were within the central part.

In July, higher chl-a concentrations spread across the lake (Figure 4). During this month, high concentration values were measured in 2002, 2003, 2006, 2009, 2010. On the other hand, 2012 and 2015, appear almost free from high chl-a values except on the southern coast.

The highest chl-a values in August were measured in 2002 (20.70 mg m^−3^), 2003 (21.61 mg m^−3^), 2006 (25.34 mg m^−3^), 2007 (18.82 mg m^−3^), and 2019 (19.76 mg m^−3^) (Figure 6). The year 2017 had very low values while 2019 had moderate to high values across the entire lake.

September–October marks the beginning of the winter cooling period causing a drop in chl-a concentrations (Figure 6 and Figure 7). The entire lake still had detectable chl-a values in September, except in 2014, 2017 and 2018. The yearly temporal and spatial distribution of water temperature across Lake Ladoga is driven mainly by the thermal bar phenomenon [1,3], which is even noticeable from satellite images [1]. The thermal bar is a common characteristic of large lakes [1]. In Lake Ladoga, the thermal bar is formed in spring and early summer [1,3]. The thermal bar is the column of water formed between the cold (deep central) and warmer (coastal) waters of a lake [17]. It is the location of vigorous water mixing caused by downwelling of water with a temperature of four degrees Celsius [3]. This is the result of the physical property of water, that has its maximum density at +4 °C, thus heavier rather than colder or warmer water, causes it to sink. The thermal bar effectively acts as a wall between the coastal and inner water masses of a lake, inhibiting mixing of the two water masses. This, in turn, only allows for near shore cyclonic currents caused by the prevalent winds and the Coriolis effect. Consequently, there is a bloom in phytoplankton biomass (chl-a producer) along the coast. The thermal bar disappears around the end of June and beginning of July when the lake’s water temperature is homogeneously above +4 °C, usually up to the depth of 50–70 m [1,3]. As the thermal bar disappears, temperature no longer plays a major role in chl-a concentrations in the lake [1,3]. The cyclonic current transports the phytoplankton to the north of Lake Ladoga. It is important to note that during August, the effect of the thermal bar has completely disappeared [1]. Before the disappearance of the thermal bar, the water flow and movement of phytoplankton is cyclonic (counterclockwise) in nature (South-East to North) [3], mainly along the shoreline (Figure 1c). The cyclonic nature of the water movement is driven by the topography, bathymetry, selective westerly winds [1], and the rotation of the earth (i.e., the so-called Coriolis phenomenon). There are also anticyclonic eddy currents in Lake Ladoga [29,30]. In general, anti-cyclonic currents are caused by weak bottom friction and southerly winds [29,30,31]. Most of the observed low chl-a concentrations are in the north and central parts of the lake while the southern coastal areas remain unchanged. From our results and in-depth literature review, the following factors were identified to be driving the on-going chl-a changes taking place in Lake Ladoga: (i) Temperature, (ii) impact from industry, external load, and littoral settlements. These coastal areas are locations of high industrial activity (e.g., paper mills), settlements and from external sources [1,3].

## 4. Discussion

As mentioned in Section 3.1 (first paragraph), we observed a gradual and negative trend in chl-a distribution, indicating a moderate improvement of water quality.

### 4.1. Effect of Temperature and Eutrophication on Chl-a Concentrations

During the month of June, high chl-a concentrations are observed only along the southern–southwestern coastal areas while the deeper parts of the lake remain clean (Figure 3), due to the presence of the thermal bar. These coastal areas have major influxes of nutrients from rivers like the Syas, Volkov and Svir, carrying municipal, industrial and agricultural waste [1,3].

The action of the southern summer winds causing anti-cyclonic eddies moves and distributes the phytoplankton mass towards the central part of the lake [1]. The temperature along the shallow coastal areas of the lake is now homogeneous [1]. Consequently, eutrophication by nutrients originating from bottom sediments could possibly be a factor contributing to chl-a concentrations [1,3]. These eutrophic sediments are thought to originate from high nutrient fall-out between the 1950s and 1990s, the era of intense eutrophication [32]. However, the present role of eutrophication of bottom sediments is arguably not a major factor [1]. Previous studies analyzing water samples from Lake Ladoga revealed that the coastal areas in the North, Southwest, and South were very eutrophic [33]. Also, the southern part of the basin has high concentrations of dissolved organic matter (DOM), primarily from external water load, excretion from green vegetation and decay of macrophytes [1].

The cooling cycle in Lake Ladoga starts in September, causing reduction in phytoplankton biomass and consequently a decline in chl-a values. An important aspect to consider here is the impact of climate change on Lake Ladoga. In general, the last two decades have been exceptionally warm, in fact, the warmest period in the entire history of climate measurement. Sharov et al. [34], reported a temperature increase of +1.5 degrees Celsius between 1950 and 2010 in Lake Ladoga [34]. Likewise, years 2000 and 2015 were exceptionally warm [18]. Consequently, this has increased the duration of the lake’s ice-free period from 210 days to 230 days [34,35,36]. A recent study by Karetnikov et al. [17], also reported increases in temperature giving rise to shorter ice periods. As a result, the increase in available sunshine (less ice) and higher surface water temperatures promote the growth of algae. A similar observation (rising water surface temperature and fewer ice days) have been reported in Lake Peipsi [37]. The impact of climate change on temperature increase promoting algal growth has also been reported in other regions as well [38,39].

### 4.2. Impact from Industries, Coastal Settlements, and External Load

The decline in chl-a (an indicator of improved water quality) is largely due to the closing of paper mills that led to less wastewater being discharged into Lake Ladoga [40]. High chl-a areas have been observed around functioning paper mills and coastal cities, these sources discharge a considerable amount of nutrient rich wastewater [5]. The closure of several mills led to a significant reduction in primary phytoplankton (chl-a) production and phosphorus concentrations [5]. The anthropogenic activities of the high-density settlements around Lake Ladoga could also be sources for nutrients driving algal blooms [41]. Also, by analyzing comprehensive water samples from Lake Ladoga, Holopainen et. al. [33], found parts of the lake to be eutrophic. They found high chl-a concentrations near Sortavala Bay (26 mg/m^3^ in August) and Pitkäranta (functioning paper mill) in the northern shores. High chl-a values were also in the southwest corner of Ladoga, in front of Zaporozhskoye (Metsäpirtti) (11.9 mg/m^3^) and in front of the city of Novaya Ladoga (20–25 mg/m^3^). The paper and pulp mills in Priozersk (Käkisalmi), Harlu and Läskelä were already established during the Finnish period (before 1945) and continued to operate during the Soviet and Russian era until their closure in the late 1980s. The Pitkäranta pulp mill was also established as early as the 1920s, and its production continues today. An active pulp mill is located near the town of Novaya Ladoga, on the south coast of Ladoga. Here a major accident occurred in 1998 when 700,000 cubic meters of toxic sludge spilled into the Syas River, about a kilometer from the shores of Lake Ladoga [5]. Phosphorus and nitrogen emissions from agriculture have also been a major nutrient source, but agriculture has declined since 1990.

External load from the large catchment area is still a major factor keeping chl-a levels elevated [6] (Figure 1b). The effect of the Volkov Bay (southern Lake Ladoga) is also obvious (Figure 3, Figure 4, Figure 5, Figure 6 and Figure 7), as the Volkov River is the largest external load on Ladoga. For example, high phosphorus concentrations of 210 μg/L were found in the 1980s as compared to 46 μg/L in the 1950s and 1960s [5]. The sources of these phosphorus values were from a large number of industrial plants (594) and agricultural enterprises (680) in the watershed [42,43]. However, due to the closure of factories in the 1990s [5,40] phosphorus concentrations in the Volkhov Bay were found to have dropped to 120 μg/l from 210 μg/l [5]. Especially the closure of paper mills has been a contributing factor to the improved water quality of Lake Ladoga [1,2,3,5].

A similar trend of water pollution has been reported at the pulp and paper mills located at the shores of Lakes Onega and Imandra [44,45,46]. Both of these locations (Kondopoga Bay and Imandra Bolshaya Bay), have seen increased nutrient loads, other toxic chemicals and degraded water quality [45]. However, in comparison to Ladoga, Onega and Imandra, are considerably cleaner (Figure 3, Figure 4, Figure 5, Figure 6 and Figure 7). To put this into perspective, the chl-a values of Lake Onega (1999–2010) in Lahti, Petrozavodsk varied between 1 and 7 mg/L without a clear trend (with an average of 2–3 mg/L), clearly indicating low pollution levels [34]. Also, Onega’s burden is only one urban center, the city of Petrozavodsk. On the other hand, recents studies in Onega have indicated significant water browning especially around Petrozavodsk Bay [47].

The pollution and degradation of water quality from wastewater discharged from pulp and paper mills, urban settlements and agricultural activities have been documented in other countries as well. For example, in Finland’s pulp and papers mills located along Lake Päijänne polluted the lake’s water, thus degrading the water quality [48].

On the other hand, it is well documented that, better waste management practices by paper mills, settlements and agricultural activities have improved the water quality of rivers and lakes [49,50]. For example, the Stora Enso Veitsiluoto Mills at Kemi, northern Finland [51,52] and the Kaukas paper mill on southern Lake Saimaa have been able to improve local water quality through better water management processes [53]. It is worthy to note that the closing of paper mills was a major factor to improved water quality in the absence of better waste management practices, e.g., [51,52,54].

## 5. Conclusions

This study assessed the trophic state of Lake Ladoga during the last 23 years (1997–2019). Geostatistical tools were used to analyze remote sensing data for this purpose. Our analysis reveals a slight decline in chl-a, suggesting there is a moderate improvement in the state of the lake. This study observed that the southernmost and shallowest part of the water body was the most problematic with high observable concentrations of chl-a. This southern area receives nutrients both from the Syas catchment and from bottom sediments causing high phytoplankton growth. Observable differences were seen in the deeper parts of Lake Ladoga in the northern part of the basin. In this northern part, reduced chl-a concentrations were observed, although there were local differences, especially on the north-eastern shore off the Pitkäranta factory site. Also, on the northern shore of Sortavala, there were exceptions as well which could be due to the discharge of municipal sewage.

It is important to note that reducing the nutrient load in Lake Ladoga due to anthropogenic activities is a slow and complicated process. These processes include effects of climate change, mainly warming and ice reduction, both of which contribute to eutrophication of the lake. The impact of climate change on rainfall is not certain, but it is generally assumed that rainfall will increase, thus, increasing the external nutrient load of Lake Ladoga. Furthermore, deforestation in the catchment area also increases nutrient leaching from the soil, and it is therefore uncertain whether the current good trend in the decrease of chl-a will continue. Despite the slight improve of the state of Lake Ladoga reported in this study, it appears that primary production is still relatively high. Our conclusion is in line with previous studies of the lake [6].

In this study, after the visual assessment of satellite imageries and an in-depth literature review, we are of the opinion that the current nutrient load and chemical waste influx in Lake Ladoga is less now, as compared to 23 years ago. We are of the opinion that to further decrease chl-a concentrations in Lake Ladoga, sewage from municipalities should be treated before entering the lake, agricultural practices have to be adjusted to reduce washing of fertilizers, the nutrient load from fish farming should be reduced and traditional fishing should be increased. These measures could potentially improve the water quality of the lake.
